# Oral Beta-Lactamase Protects the Canine Gut Microbiome from Oral Amoxicillin-Mediated Damage

**DOI:** 10.3390/microorganisms7050150

**Published:** 2019-05-27

**Authors:** Sheila Connelly, Brian Fanelli, Nur A. Hasan, Rita R. Colwell, Michael Kaleko

**Affiliations:** 1 Synthetic Biologics, Inc., 9605 Medical Center Drive, Suite 270, Rockville, MD 20850, USA; mkaleko@syntheticbiologics.com; 2 CosmosID, Inc., Rockville, MD 20850, USA; brian.fanelli@cosmosid.com (B.F.); nur.hasan@cosmosid.com (N.A.H.); rita.colwell@cosmosid.com (R.R.C.); 3 University of Maryland Institute of Advanced Computer Studies, College Park, MD 20740, USA

**Keywords:** beta-lactamase, gut microbiome, antibiotic resistance

## Abstract

Antibiotics damage the gut microbiome, which can result in overgrowth of pathogenic microorganisms and emergence of antibiotic resistance. Inactivation of antibiotics in the small intestine represents a novel strategy to protect the colonic microbiota. SYN-004 (ribaxamase) is a beta-lactamase formulated for oral delivery intended to degrade intravenously administered beta-lactam antibiotics in the gastrointestinal (GI) tract. The enteric coating of ribaxamase protects the enzyme from stomach acid and mediates pH-dependent release in the upper small intestine, the site of antibiotic biliary excretion. Clinical benefit was established in animal and human studies in which ribaxamase was shown to degrade ceftriaxone in the GI tract, thereby preserving the gut microbiome, significantly reducing *Clostridioides difficile* disease, and attenuating antibiotic resistance. To expand ribaxamase utility to oral beta-lactams, delayed release formulations of ribaxamase, SYN-007, were engineered to allow enzyme release in the lower small intestine, distal to the site of oral antibiotic absorption. Based on in vitro dissolution profiles, three SYN-007 formulations were selected for evaluation in a canine model of antibiotic-mediated gut dysbiosis. Dogs received amoxicillin (40 mg/kg, PO, TID) +/- SYN-007 (10 mg, PO, TID) for five days. Serum amoxicillin levels were measured after the first and last antibiotic doses and gut microbiomes were evaluated using whole genome shotgun sequence metagenomics analyses of fecal DNA prior to and after antibiotic treatment. Serum amoxicillin levels did not significantly differ +/- SYN-007 after the first dose for all SYN-007 formulations, while only one SYN-007 formulation did not significantly reduce systemic antibiotic concentrations after the last dose. Gut microbiomes of animals receiving amoxicillin alone displayed significant loss of diversity and emergence of antibiotic resistance genes. In contrast, for animals receiving amoxicillin + SYN-007, microbiome diversities were not altered significantly and the presence of antibiotic resistance genes was reduced. These data demonstrate that SYN-007 diminishes amoxicillin-mediated microbiome disruption and mitigates emergence and propagation of antibiotic resistance genes without interfering with antibiotic systemic absorption. Thus, SYN-007 has the potential to protect the gut microbiome by inactivation of beta-lactam antibiotics when administered by both oral and parenteral routes and to reduce emergence of antibiotic-resistant pathogens.

## 1. Introduction

The gut microbiome comprises genomes of the microbiota inhabiting the gastrointestinal (GI) tract and works symbiotically with the host to maintain health. Antibiotics can disrupt this complex ecosystem, causing dysbiosis, an alteration of the normal microbial balance. Antibiotic-mediated dysbiosis can interfere with host resistance to colonization by opportunistic pathogens, notably, *Clostridioides difficile* and vancomycin-resistant *Enterococcus* spp. (VRE), and also result in emergence of antibiotic resistance. Broad-spectrum antibiotics, of which the beta-lactams are the most commonly used, are especially damaging [1,2]. Concordantly, beta-lactams were the only drug significantly linked to gut microbiome disruption in a comprehensive phenotype-controlled microbiome clinical study [3], and have been associated with an increased risk of *C. difficile* infection (CDI) [4,5]. Therefore, a useful strategy to protect the gut microbiome from the adverse effects of beta-lactams is to limit exposure of the microbiota in the colon to antibiotics, without affecting the efficacy of the antibiotics in treating the infection.

Use of beta-lactamases, enzymes that specifically hydrolyze and inactivate beta-lactam antibiotics, to degrade beta-lactams in the GI tract represents a novel strategy for preservation of the gut microbiome. Proof of concept for this approach was achieved in both in animal models and human clinical trials with SYN-004 (ribaxamase), an orally administered beta-lactamase, by demonstrating degradation of intravenously (IV) administered ceftriaxone in the GI tract, protection of the gut microbiome from antibiotic-mediated alteration, and reduction of emergence of antibiotic resistance [6,7,8]. In a phase 2b clinical study, ribaxamase significantly reduced CDI in patients treated with IV ceftriaxone for a lower respiratory tract infection, without compromising pulmonary infection control [7,9].

Ribaxamase, a class A serine beta-lactamase, was engineered specifically to degrade most penicillins and cephalosporins [10] and is intended for oral administration with IV beta-lactam antibiotics, many of which are excreted via bile into the upper small intestine. The clinical ribaxamase formulation consists of enteric-coated enzyme pellets formulated for pH-mediated release in the duodenum and upper small intestine [11]. While ribaxamase has demonstrated clinical benefit in protecting the gut microbiome from damage caused by IV antibiotics, the vast majority of beta-lactams are administered orally [12] and the current ribaxamase formulation is not suitable for use with oral antibiotics. Orally delivered beta-lactams, such as amoxicillin, are absorbed from the proximal small intestine [13], which is also the site of ribaxamase release. Therefore, administration of the current ribaxamase formulation with oral antibiotics is predicted to interfere with antibiotic systemic absorption due to ribaxamase-mediated degradation in the small intestine.

To expand applications of ribaxamase to include the oral beta-lactams, new oral formulations were engineered specifically for site-specific enzyme release in the GI tract. The predicted ideal location for beta-lactamase activity is distal to the site of antibiotic uptake, to not interfere with systemic absorption, and proximal to the colon, to ensure antibiotic inactivation prior to reaching and harming the colonic microbiota. Therefore, the ileum and/or ileocecal junction was targeted for beta-lactamase release. Several approaches, including bacterially activated, timed/sustained release, and pH-sensitive release formulations were successful in targeting drug delivery to the GI tract following oral administration [14,15]. Bacterially activated systems may be the most selective relying on enzymatic degradation of polysaccharides, such as pectin, chitosan, and cellulose, by microbiota present in the colon, therefore targeting release within the colon. However, delaying antibiotic degradation to within the colon is expected to be less efficient in protecting the colonic microbiota than inactivation in the distal small intestine. Alternatively, timed and sustained-release systems are useful in some instances, but they are dependent upon GI transit times, which can be extremely variable [14], making these formulations potentially unreliable for use with oral antibiotics. In contrast, pH-based systems employ ionic polymers responsive to low (cationic) or high (anionic) pH [15] and affect site-specific drug delivery by exploiting the pH gradient along the GI tract. The pH of the mammalian GI tract progressively increases from the acidic stomach (pH 1.0–3.5) to pH 5.0–6.0 in the duodenum and proximal small intestine, to a peak of pH 7.0–8.0 in the ileum in the distal small intestine, decreasing again to pH 5.0–6.0 in the cecum [16,17]. Such pH-mediated release systems have been shown to direct reliable drug delivery to specific sites along the GI tract, including the duodenum and ileum [16,18].

Indeed, successful targeting ribaxamase release to the upper small intestine has been confirmed clinically, where ribaxamase was shown to be present and functional in human intestinal chyme [8]. The pH-triggered ribaxamase formulation employs the anionic methacrylate polymer, EUDRAGIT^®^ L30-D55 (Evonik Industries AG, Essen, Germany) [19], coating sucrose pellets layered with the enzyme [11]. The L30-D55 coating remains intact at low, gastric pH, thus protecting the enzyme from acidic conditions of the stomach and dissolving rapidly to release the enzyme at pH ≥5.5, the pH of the duodenum and upper small intestine [11]. Furthermore, EUDRAGIT^®^ polymers can be engineered to dissolve at a selected pH, including pH >7.0, for release in the ileum [16,18]. Therefore, a pH-mediated release strategy was employed to generate new ribaxamase formulations intended for use with oral beta-lactam antibiotics.

Distal release formulations of ribaxamase, SYN-007, were manufactured for pH-mediated release in the lower small intestine. Three SYN-007 formulations with favorable in vitro dissolution profiles were evaluated in a canine model of oral amoxicillin-mediated gut dysbiosis. Dogs were selected for study because the dimensions, motility, and absorption characteristics of the canine small intestine are similar in humans [20], as is composition of the gut microbiome [21]. Animals received oral amoxicillin +/- SYN-007 for five days. Serum antibiotic levels were measured after the first and last doses to assess SYN-007 interference with amoxicillin systemic absorption. Metagenomics analyses were performed using fecal DNA collected prior to and following antibiotic administration to evaluate antibiotic-mediated changes in the gut microbiome and gut resistome.

## 2. Materials and Methods

### 2.1. Test Article

Three formulations of SYN-007 were prepared (Table 1). Sugar spheres layered with ribaxamase by spray application [11] were used as the starting material for all formulations. Formulation 1 consisted of ribaxamase layered sugar pellets coated with Eudragit^®^ FS30D (Evonik, Essen, Germany) at a 20% polymer weight gain via spray application using a fluid bed Mini-coater/Drier (Caleva Process Solutions Limited, Dorset, England). Size 9h gelatin capsules (2.69 mm diameter × 5.1 mm length; Torpac, Fairfield, NJ, USA) were loaded with 12 FS30D coated ribaxamase pellets. Filled 9h capsules were banded and spray coated with FS30D at 6.3 mg/cm^3^ polymer weight gain. Five of these filled and coated 9h capsules were loaded into one size 0 uncoated hard capsule for a total ribaxamase dose of 9.4 mg/size 0 capsule. Formulation 2 consisted of 64 FS30D coated ribaxamase pellets loaded into an uncoated size 5 hard capsule for a total ribaxamase dose of 10 mg/size 5 capsule. Formulation 3 consisted of ribaxamase layered sugar pellets coated with Eudragit^®^ L30-D55 (Evonik, Essen, Germany) at a 21% polymer weight gain via spray application as described [11]. Size 9h capsules were loaded with 8 L30-D55 coated ribaxamase pellets. Filled 9h capsules were banded and spray coated with FS30D at 6.3 mg/cm^3^ polymer weight gain. Eight filled and coated 9h capsules were loaded into one size 0 uncoated hard capsule for a total ribaxamase dose of 10 mg/size 0 capsule. SYN-007 formulations were manufactured and tested by Aptuit, LLC (formerly Kuecept, Ltd, London, UK).

### 2.2. In Vitro Dissolution Analyses

The three SYN-007 formulations were evaluated for beta-lactamase release under differing pH conditions. Formulations were exposed to an HCl solution (0.1N) of pH 1.1 for 2 hours, after which the pH was increased to pH 5.5 (0.1N HCl, 0.3M K_2_HPO_4_, pH adjusted to 5.5) for 2 hours, and then to pH 7.1 (0.1N HCl, 0.3M K_2_HPO_4_, pH adjusted to 7.1) for 4 hours. Samples were collected every hour and assessed for percent beta-lactamase release as determined by 0.2 µm filtration followed by UV absorbance at 280 nm subtracted from absorbance at 260 nm (A_280_–A_260_). Analyses were performed by Aptuit LLC (formerly Kuecept, Ltd, London, UK).

### 2.3. Animals and Test Article Administration

Twenty healthy adult (7 to 8 months old) female Beagle dogs, 6.5 to 7.7 kg, were obtained from Covance Research Products (Denver, PA, USA). Animals were naïve and had not been exposed to antibiotics previously. After arrival to the test site, Calvert Laboratories, Inc. (Scott Township, PA, USA), animals were acclimated to their new environment/housing for 24 days prior to their first fecal collection during which time the health status of each animal was evaluated daily. Dogs were individually housed in compliance with USDA Guidelines and were permitted to comingle except on feces collection days and on study days 1–6. Animals were fed antibiotic-free PMI® Canine Diet (PMI Nutrition Pet Food, Land O’Lakes, Inc., Arden Hills, MN, USA). Animals were randomly divided into 4 cohorts (*n* = 5 each). The cohorts were: Amoxicillin alone, Amoxicillin + SYN-007 Formulation 1, Amoxicillin + SYN-007 Formulation 2, and Amoxicillin + SYN-007 Formulation 3.

On study days 1–5, food was presented three times per day, 1.5 hours after each of amoxicillin +/- SYN-007 dose. On study day 6, animals were presented with food 1.5 hours after the last dose of amoxicillin +/- SYN-007. Dogs had free access to water at all times. Body weights of each animal were recorded daily on study days 1–6 and used to calculate the volume of amoxicillin administered each day at 40 mg/kg per dose.

Amoxicillin was supplied as a fruit-flavored powder (4000 mg/bottle, Sandoz, Holzkirchen, Germany, NDC 0781–6157–52) and was suspended in 100% Mott’s apple juice (pH 3.0) instead of water, per package instructions, at 400 mg/5 mL. Animals received amoxicillin orally (40 mg/kg), three times a day at 8 hours intervals, with or without one capsule of SYN-007 (Formulations 1, 2 or 3), with the last dose on the morning of day 6, for a total of 16 doses of amoxicillin +/- SYN-007. Each amoxicillin dose was followed by 5 mL Mott’s apple juice delivered orally with a 10 mL plastic syringe. After all animals received amoxicillin, appropriate animals received one capsule of SYN-007 orally followed by another 5 mL of apple juice to ensure that the SYN-007 capsule was swallowed. The use of apple juice as the antibiotic diluent and additional apple juice after oral antibiotic and SYN-007 administration ensured pH of the stomach remained acidic to prevent premature release of the beta-lactamase from the enteric formulations.

Dogs were bled after the first dose on day 1 and on day 6 at 0.5, 1, 2, 3, 4, 6, and 8 hours after amoxicillin administration. Approximately 2 mL whole blood samples were obtained via direct venipuncture of a jugular vein using Gold-Top SST blood collection tubes (BD Vacutainer®, Becton Dickinson and Company, Franklin Lakes, NJ, USA). Following collection, blood samples were retained at room temperature for ~10 minutes to clot, and centrifuged to separate the serum as per package directions. Serum was stored at −80°C. Fecal samples were collected twice, on day -1 prior to antibiotic dosing and on day 6 following antibiotic +/- SYN-007 dosing. Samples were collected fresh upon defecation and placed directly into the OMNIgene® GUT sample kit collection tubes (DNA Genotek, Ottawa, Canada). Fecal samples were stored at room temperature.

All animal procedures were conducted in accordance with principles and guidelines established by the Calvert Institutional Animal Care and Use Committee (IACUC) in accordance with the Animal Welfare Act at Calvert Laboratories, Inc. (Scott Township, PA, USA). The animal study protocol (Study No. 0832DS123.001) was approved by the Calvert IACUC. Calvert Laboratories, Inc. is fully accredited by the Association for Assessment and Accreditation of Laboratory Animal Care (AALAC).

### 2.4. Amoxicillin Serum Measurement

Serum was analyzed for amoxicillin using liquid chromatography turbo ion spray tandem mass spectrometry (LC/MS/MS) following protein precipitation extraction using Applied Biosystems Triple Quad API 5000 LC/MS/MS system with Turbo ion spray interfaces (Applied Biosystems Company, Foster City, CA, USA). The negative ions were measured in MRM mode. The data were acquired and analyzed by Applied Biosystems “Analyst” software, version 1.6. The lower limit of quantitation (LLOQ) was 101.7 ng/mL and the upper limit of quantitation (ULOQ) was 20334.2 ng/mL. A calibration curve composed of blanks, two zero standards, and 10 nonzero calibration standards covering a range of 101.7 to 20334.2 ng/mL were analyzed with the samples. Quality control samples at three different concentration levels, corresponding to 300.2 ng/mL, 7503.9 ng/mL, and 15007.8 ng/mL, were analyzed with the samples. Analyte to internal standard peak area ratio values were used to construct the calibration curve and to determine sample concentrations. Linear regression with 1/x^2^ weighting was used to obtain the best fit of the data for the calibration curve. The overall % accuracy of the quality control samples ranged from 97.0–102.6%. Inter-assay precision was determined by the % CV of the quality control samples and ranged from 3.6–4.3%. Amoxicillin serum analysis assay was developed, validated, and performed by Sannova Analytical, Inc. (Somerset, NJ, USA). Area under the curve calculations and statistical analyses were performed using GraphPad Prism 7 (GraphPad Company, San Diego, CA, USA).

### 2.5. Fecal DNA Extraction, Whole Genome Shotgun Sequencing and Metagenomic Analyses

Total DNA was isolated from fecal specimens, using MOBIO Power-Soil® DNA Isolation Kit (Qiagen, Germantown, MD, USA), following manufacturer’s instructions. Each DNA sample was normalized in 3–18 µL of nuclease-free water to a final concentration of 0.5 ng µL^−1^ using Biomek FX liquid handler (Beckman Coulter Life Sciences, Brea, CA, USA). Libraries were constructed using Nextera XT Library Prep Kit (Illumine, San Diego, CA, USA). For each sample, an input of 0.5 ng was used in the tagmentation reaction, followed by 13 cycles of PCR amplification using Nextera i7 and i5 index primers and 2X KAPA master mix per the modified Nextera XT protocol. The PCR products were purified using 1.0X speed beads and eluted in 15 uL of nuclease-free water. Final libraries were quantified by PicoGreen fluorometric assay (100X final dilution) and concentrations were in the range of 0.1–4.0 ng uL^−1^. The libraries were pooled by adding an equimolar ratio of each based on the concentration determined by PicoGreen, and loaded onto a high sensitivity (HS) chip run on the Caliper LabChipGX (Perkin Elmer, Waltham, MA, USA). The base pair size reported was in the range of 301–680 bp. Samples were sequenced using a single Illumina HiSeq v3 flowcell by multiplexing eight libraries per lane targeting 25 million 100 bp reads per sample. Standard read quality assessments were performed prior to metagenomics analyses using open source BBDuk software from BBTools (https://jgi.doe.gov/data-and-tools/) and all samples conformed to an average read quality of Q20 indicating 99% sequencing accuracy (https://www.illumina.com/science/education/sequencing-quality-scores.html). Reads per sample were consistent, 35,000,000 ± 14,000,000 reads/sample (median 32,000,000), indicating comparable read depth.

Unassembled whole genome shotgun metagenomic sequencing reads were directly analyzed using the CosmosID, Inc. bioinformatics software package (CosmosID Inc., Rockville, MD, USA), as described [22,23,24,25], to achieve bacterial identification to species, subspecies, and/or strain level and quantification of microorganism relative abundance. Briefly, the system utilizes a high performance data-mining k-mer algorithm and highly curated dynamic comparator databases (GeneBook®, CosmosID, Inc., Rockville, MD, USA) that rapidly disambiguate millions of short reads into the discrete genomes or genes engendering the particular sequences. The GeneBook^®^ databases are composed of over 150,000 microbial genomes and gene sequences representing over 1000 bacterial, 5000 viral, 250 protists and 1500 fungal species, as well as over 5500 antibiotic resistant and virulence associated genes. Each GeneBook^®^ database was screened and cleaned for host genome sequences including human, pig, and dog genomes, followed by validation by analyzing each host genome as a query in the curated databases. The web portal is hosted at AWS cloud and can be accessed at https://app.cosmosid.com/login.

Metagenomic analysis is based on a proprietary high performance data-mining k-mer algorithm, implemented by C, as the core engine. The analysis algorithm has two separable comparators: a pre-computation phase for the reference database and a per-sample computation. The input to the pre-computation phase is a reference microbial genome or antibiotic resistance GeneBook^®^ database, and its output is phylogeny trees, together with sets of variable length k-mer fingerprints (biomarkers) that are uniquely identified with distinct nodes, creating branches and leaves of the tree. The reference GeneBook^®^ database constitutes both publicly available genomes or gene sequences, such as NCBI- RefSeq/WGS/SRA/nr, PATRIC, M5NR, IMG, ENA, DDBJ, CARD, ResFinder, ARDB, ARG-ANNOT, mvirdb, VFDB, as well as a subset of genomes sequenced by CosmosID, Inc. and its collaborators. The second per-sample computational phase searches the hundreds of millions of short sequence reads or contigs from draft assembly against the fingerprint sets. The resulting statistics are analyzed to give fine-grain composition and relative abundance estimates. Edit distance-scoring techniques are used to compare a target genome or gene with the reference set. The algorithm provides similar functionality as BLAST. Classification precision is maintained employing aggregation statistics. Enhanced detection specificity is achieved by running comparators in sequence. In summary, the two-part analysis consists of first finding reads in which there is an exact match with a k-mer uniquely identified with a GeneBook^®^ reference database, and then statistically scoring the entire read against the GeneBook^®^ reference to verify the read is indeed uniquely identified with that reference. For each sample, the reads from a species are assigned to a strain with the highest aggregation statistics. Similarly, the community resistome, the collection of antibiotic resistance genes in the microbiome, was also identified using the CosmosID, Inc. bioinformatics software package to query unassembled sequence reads against the CosmosID, Inc. curated antibiotic resistance gene database in a manner analogous to the bacterial species identification.

Analyses of the bacterial sequence data included Shannon alpha diversity [26], principal component analyses, stacked bar graphs, and heatmaps based on relative abundance of each microorganism (%) in each sample using the NMF R software package [27]. Resistome analysis was performed by identification of antibiotic-resistance genes based on percentage of gene coverage for each gene as a function of the gene-specific read frequency in each sample. Statistical analyses were performed using Microsoft Excel 2016 (Microsoft Corporation, Redmond, WA, USA) or GraphPad Prism 7 (GraphPad Company, San Diego, CA, USA).

### 2.6. Data Availability

Fecal DNA metagenomics sequencing data are available in Sequence Read Archive (SRA) (https://submit.ncbi.nlm.nih.gov/subs/sra/), Accession SRP093227.

### 2.7. Ethics Approval

All animal procedures were approved by and conducted in accordance with principles and guidelines established by the Calvert Institutional Animal Care and Use Committee (IACUC) in accordance with the Animal Welfare Act at Calvert Laboratories, Inc. (Scott, PA, USA). The animal study protocol (Study No. 0832DS123.001) was approved by the Calvert IACUC. Calvert Laboratories, Inc. is fully accredited by the Association for Assessment and Accreditation of Laboratory Animal Care (AAALAC).

## 3. Results

### 3.1. SYN-007 Exhibits pH-Dependent Dissolution In Vitro

Three SYN-007 formulations were manufactured (Table 1). All formulations consisted of sugar spheres layered with the beta-lactamase enzyme, ribaxamase, coated with methacrylic acid polymers, Eudragit®. The enteric coatings, Eudragit^®^ L30-D55 and Eudragit^®^ FS30D, engineered to remain intact at low pH and dissolve at pH > 5.5 and pH > 7.0, respectively [19], are intended to protect the enzyme from stomach acid and to release beta-lactamase as the pH progressively increases during transit of the GI tract [16,17]. Formulation 1 was composed of ribaxamase pellets coated with Eudragit^®^ FS30D loaded into small, size 9h capsules that were also coated with FS30D. Formulation 2 was manufactured as FS30D-coated enzyme pellets encapsulated into larger, uncoated size 5 hard capsules. Formulation 3 consisted of Eudragit^®^ L30-D55 coated ribaxamase pellets [11] within FS30D-coated size 9h capsules. Formulations 1 and 3 were delivered within size 0 uncoated capsules for ease of administration.

**Figure 1:**
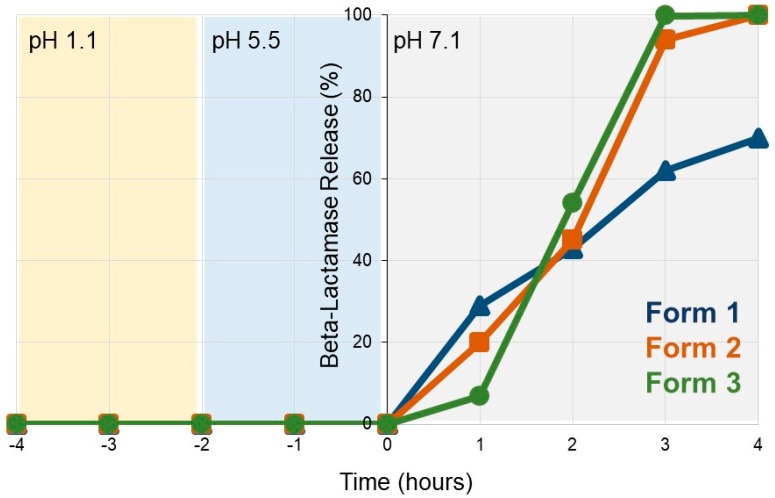
SYN-007 in vitro dissolution profiles. SYN-007 formulations were incubated at pH 1.1 for 2 hours, then pH 5.5 for 2 hours, and pH 7.1 for 4 hours. Samples were collected every hour and assessed for beta-lactamase release (%) using UV absorption (A_280_–A_260_). Blue triangles, SYN-007 Formulation 1, orange squares, SYN-007 Formulation 2, green circles, SYN-007 Formulation 3.

To verify pH-based dissolution, the SYN-007 formulations were incubated first in a pH 1.1 solution for 2 hours which simulates the acidic environment of the stomach and tests the enteric coating for protection of the enzyme. This incubation was followed by 2 hours at pH 5.5 followed by an additional 4 hours at pH 7.1 (Figure 1). No enzyme release was detected during pH 1.1 and pH 5.5 incubations indicating that the FS30D enteric coating remained intact under these conditions for all three formulations. Upon incubation in pH 7.1 buffer, enzyme release initiated within the first hour, with Formulation 1 showing 29% release, Formulation 2, 20%, and Formulation 3, 7% at 1 hour. Release kinetics were similar for all formulations at 2 hours with complete dissolution of Formulation 3 occurring by 3 hours and Formulation 2 by 4 hours at pH 7.1. In contrast, incomplete, 70% release of the beta-lactamase was detected with Formulation 1 after 4 hours at pH 7.1. Therefore, the three SYN-007 formulations displayed differing pH-mediated dissolution kinetics in vitro.

### 3.2. SYN-007 Formulation 3 Did Not Significantly Affect Oral Amoxicillin Systemic Absorption

**Figure 2:**
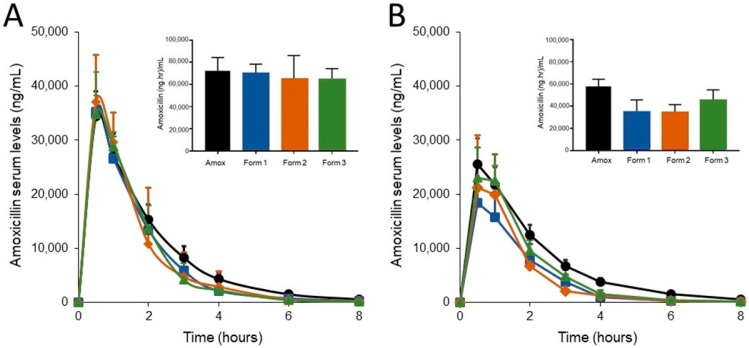
Amoxicillin serum levels. Amoxicillin was measured in dog serum collected at the indicated times. (**A**) Amoxicillin levels after the first dose of amoxicillin (Day 1) +/- SYN-007 (Formulations 1, 2 or 3). Inset bar graph displays the area under the curve (AUC) for each group. (**B**) Amoxicillin levels after the last (16^th^) dose of amoxicillin (Day 6) +/- SYN-007. Inset bar graph displays the AUC for each group. Black, Amoxicillin alone; blue, Amoxicillin + Formulation 1, orange, Amoxicillin + Formulation 2, green, Amoxicillin + Formulation 3. Data are displayed as mean + standard deviation (*n* = 5). *p* values were obtained by analysis of the AUC from each group for each collection day using Kruskal-Wallis non-parametric ANOVA with Dunn’s multiple comparisons test (Graphpad Prism 7) comparing each group to amoxicillin alone. Day 1: Amoxicillin vs. Formulation 1, *p* > 0.9999, Amoxicillin vs. Formulation 2, *p* = 0.2925, and Amoxicillin vs. Formulation 3, *p* = 0.8551. Day 6: Amoxicillin vs. Formulation 1, *p* = 0.0083, Amoxicillin vs. Formulation 2, *p* = 0.0117, Amoxicillin vs. Formulation 3, *p* = 0.4034.

The SYN-007 formulations were evaluated with oral amoxicillin administration in dogs. Animals were administered oral amoxicillin three times a day for five days with their final dose on the morning of day 6, for a total of 16 doses. Cohorts of animals also received SYN-007 Formulation 1, 2 or 3 orally, immediately following oral amoxicillin administration, three times a day for 16 total doses. To evaluate amoxicillin systemic levels, serum was collected from each animal following the first dose of amoxicillin +/- SYN-007 on day 1 and the last dose on day 6. Amoxicillin serum levels were analyzed by comparing the area under the curve (AUC) for each SYN-007 cohort to the AUC for the amoxicillin alone group for each day (Figure 2). On day 1, there was no significant difference in amoxicillin serum level AUC for any cohort (Figure 2A). However, by day 6, after 16 doses of amoxicillin + SYN-007, significantly decreased amoxicillin serum level AUC was observed for both amoxicillin + Formulation 1 (*p* = 0.008) and amoxicillin + Formulation 2 (*p* = 0.012) cohorts compared to amoxicillin alone (Figure 2B). These data suggest that Formulations 1 and 2 were released prematurely in the small intestine and degraded the amoxicillin prior to its systemic absorption. In contrast, amoxicillin serum level AUC was not significantly different (*p* = 0.403) for animals that received amoxicillin + Formulation 3. Therefore, Formulation 3 was the only beta-lactamase preparation that did not significantly affect oral amoxicillin systemic absorption.

### 3.3. SYN-007 Mitigated Oral Amoxicillin-Mediated Microbiome Damage

**Figure 3:**
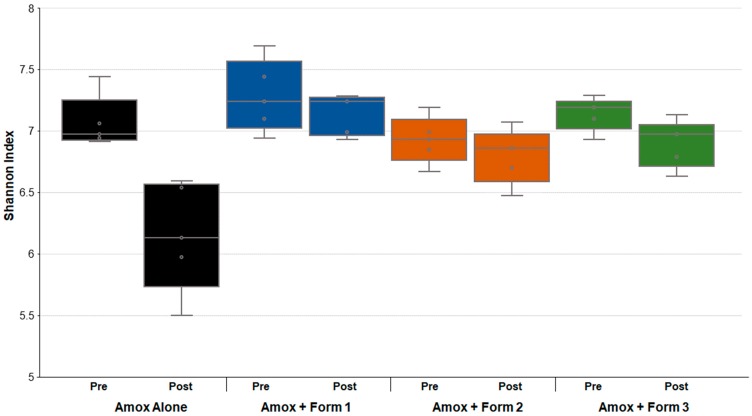
Comparison of dog fecal microbiome Shannon alpha diversity prior to and after amoxicillin treatment. Fecal microbiome metagenomics data were analyzed by Shannon index and are displayed for each cohort (*n* = 5) as box plots. *p* values were obtained by comparing pretreatment Shannon indexes (Pre) to post-treatment Shannon indexes (Post) of each cohort using Kruskal-Wallis non-parametric ANOVA with Dunn’s Multiple Comparisons test (Graphpad Prism 7). Black, Amoxicillin alone, *p* < 0.0271; blue, Amoxicillin + SYN-007 Formulation 1, *p* > 0.9999; orange, Amoxicillin + SYN-007 Formulation 2, *p* > 0.9999; green, Amoxicillin + SYN-007 Formulation 3, *p* = 0.5604.

To assess changes in the gut microbiomes of the dogs, DNA was extracted from feces collected prior to and after antibiotic +/- SYN-007 treatment and subjected to whole genome shotgun metagenomic analyses. Relative abundance of each bacterial strain in each sample for each animal was determined to allow microbiome composition comparisons. Prior to amoxicillin exposure, microbiomes of all groups displayed similar species richness with 307 ± 46 bacterial species identified in pretreatment microbiomes with no significant difference between cohorts (0.288 ≤ *p* ≤ 0.999). Following amoxicillin administration, microbiomes from animals exposed to amoxicillin alone displayed a significant decrease in bacterial species richness (*p* = 0.006), while no significant decrease in species abundance was observed in the presence of SYN-007 (Table 2). Shannon alpha diversities were calculated for each sample pre and post-treatment (Figure 3). Amoxicillin alone resulted in a significantly lower Shannon index, compared to pretreatment (*p* = 0.027). In contrast, Shannon indices were not significantly different pre and post-treatment for all amoxicillin + SYN-007 cohorts. These species richness and alpha diversity data indicate that all SYN-007 formulations protected the gut microbiota from amoxicillin-mediated alteration.

**Figure 4:**
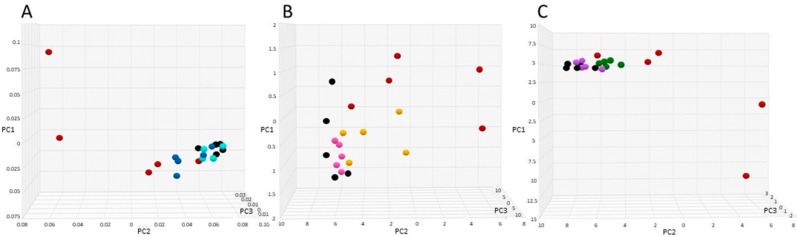
Principal component analyses of fecal microbiomes. Fecal microbiomes for each animal at each time point were analyzed via principal component analyses. (**A**) Amoxicillin alone vs. Amoxicillin + Formulation 1, black, amoxicillin pretreatment, red, amoxicillin post treatment, light blue, Amoxicillin + Formulation 1 pretreatment, dark blue, Amoxicillin + Formulation 1, post-treatment. (**B**) Amoxicillin alone vs. Amoxicillin + Formulation 2, black, amoxicillin pretreatment, red, amoxicillin post-treatment, pink, Amoxicillin + Formulation 2 pretreatment, yellow, Amoxicillin + Formulation 2 post-treatment. (**C**) Amoxicillin alone vs. Amoxicillin + Formulation 3, black, amoxicillin pretreatment, red, amoxicillin post-treatment, purple, Amoxicillin + Formulation 3 pretreatment, green, Amoxicillin + Formulation 3 post-treatment.

Principle component analysis (PCA) was performed to compare pretreatment microbiome composition to post-treatment. Distance between points indicates degree of difference in sample composition with points closer together reflecting more similarity. For ease of assessment, amoxicillin alone was compared to each amoxicillin + SYN-007 group in separate PCAs (Figure 4), and all samples were included in additional PCAs (Figures S1 and S2). In each case, pretreatment and amoxicillin + SYN-007 samples clustered more closely together than amoxicillin alone post-treatment samples. Amoxicillin + Formulation 1 (Figure 4A) and Amoxicillin + Formulation 3 (Figure 4C) post-treatment microbiomes appeared more similar to pretreatment than amoxicillin + Formulation 2 microbiomes (Figure 4B). A pretreatment sample from the amoxicillin + Formulation 1 cohort (dog 9) was concluded to be an outlier and omitted from the PCA displayed in Figure 4A and Supplemental Figure S1. For comparison, this sample was included in the PCA results displayed as Supplemental Figures S2 and S3.

**Figure 5:**
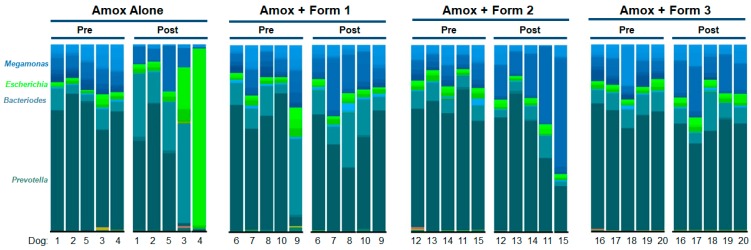
Species level stacked bar graph of fecal microbiomes. Fecal microbiomes for each animal at each time point were analyzed via stacked bar graph comparing pretreatment to post-treatment. The genera of abundant species are displayed on the left, animal numbers are displayed on the bottom, and treatment groups and collection time point displayed at the top.

**Figure 6:**
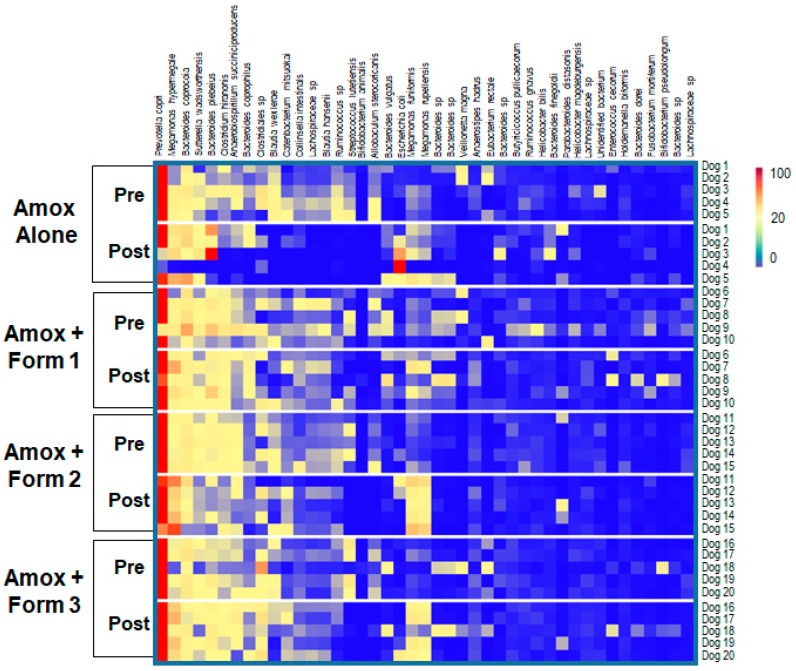
Heatmap analysis of the relative abundance of selected bacterial species present in the dog fecal microbiomes. Species composition of fecal microbiomes of animals in each treatment cohort are displayed as the abundance of each bacterial species relative to all species in each fecal sample. Each row represents an individual animal at the indicated time point. Bacterial taxa are indicated at the top of the figure, cohort and collection day are indicated on the left, and animal numbers displayed on the right. The color gradient key displays a linear scale of relative abundance.

To visualize specific changes in the canine microbiomes, stacked bar graphs (Figure 5) and heatmaps (Figure 6) of bacterial taxa were constructed, based on relative abundance of each bacterial species in each sample for each animal and organized to allow comparison of microbiomes of animals before and after treatment with amoxicillin +/- SYN-007. Compared to pretreatment microbiomes, amoxicillin alone resulted in a reduction and/or loss of specific bacterial species and overgrowth of other taxa, while amoxicillin + SYN-007 microbiomes displayed fewer alterations in microbiota composition. Overgrowth of *E. coli* was observed following amoxicillin exposure in dogs 3 and 4 of the amoxicillin alone cohort, and was not observed in any SYN-007 cohort. In contrast, increased abundance of *Megamonas hypermegale* was observed in all SYN-007 cohorts, especially noteworthy in dogs 11 and 15 in the amoxicillin + Formulation 2 cohort after treatment. Reduced abundance of several genera, including *Blautia* and *Ruminoccocus,* in the amoxicillin alone cohort was not observed with SYN-007. These data demonstrate that amoxicillin caused major alterations in gut microbiome composition that were attenuated with SYN-007.

### 3.4. SYN-007 Reduced Antibiotic Resistance Gene Propagation

**Figure 7:**
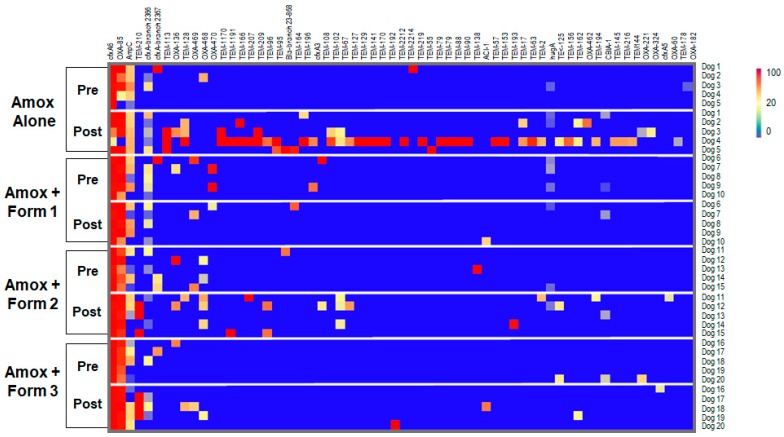
Heatmap analysis of the frequency of beta-lactamase genes in the dog fecal microbiomes. Fecal microbiome metagenomics data were analyzed for the presence of antibiotic resistance genes based on the percentage gene coverage as a measure of relative gene frequency in each sample. Each row represents an individual animal at the indicated collection day (pre or post treatment). Beta-lactamase genes are identified at the top, treatment group and day of fecal collection on the left, and the animal numbers on the right. The color gradient key displays a linear scale of the percentage gene coverage as a measure of relative gene frequency.

To determine if SYN-007 affected antibiotic resistance, fecal DNA metagenomics data were analyzed for presence of antibiotic resistance genes as a measure of the population of antibiotic resistant bacteria in the gut microbiome. To visualize specific changes in the resistomes, heatmaps of antibiotic resistance genes in the fecal microbiome of each animal before and after antibiotic treatment were generated (Figure 7 and Figure S4). Several beta-lactamase genes, genes conferring resistance specifically to beta-lactam antibiotics, were observed following amoxicillin exposure (Figure 7). The amoxicillin alone cohort displayed the most beta-lactamase genes post treatment, consisting of genes encoding class A TEM beta-lactamases and several class D OXA beta-lactamases. Amoxicillin + Formulation 2 microbiomes contained more beta-lactamase genes post treatment than Amoxicillin + Formulation 1 and Formulation 3 cohorts.

**Figure 8:**
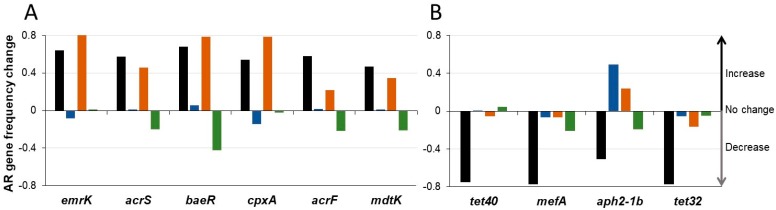
Changes in the frequency of selected antibiotic resistance genes. Change in relative frequency (mean) of indicated antibiotic resistance genes for each cohort from pretreatment to post-treatment is displayed. A negative value indicates reduction in frequency, positive value indicates increased frequency, and zero value represents no change in gene frequency. (**A**) Selected antibiotic resistance genes that increased in frequency in the Amoxicillin alone cohort. (**B**) Selected antibiotic resistance genes that decreased frequency in the Amoxicillin alone cohort. Black, Amoxicillin alone, blue, Amoxicillin + Formulation 1, orange, Amoxicillin + Formulation 2, and green, Amoxicillin + Formulation 3.

In addition to beta-lactamases, other resistance gene frequencies were affected by antibiotic exposure (Figure 8 and Figure S4). Two patterns of resistance gene frequency alterations were observed; those that increased with exposure to amoxicillin alone (Figure 8A), and those that displayed reduced frequency after antibiotic alone exposure (Figure 8B). Many of the genes displaying increased frequency post-amoxicillin alone encode components of multidrug efflux transporter systems, systems that confer resistance to a broad range of antibiotics. Notably, amoxicillin + Formulation 2 resistomes displayed an increase in these genes similar to the amoxicillin alone cohort, while amoxicillin + Formulation 1 or 3 resistomes were a relatively unchanged or decreased frequency of these genes, respectively (Figure 8A). In contrast, genes displaying decreased frequency in amoxicillin alone resistomes confer resistance to specific antibiotic classes, such as aminoglycosides (*aph2–1b*), macrolides (*mefA*), and tetracyclines (*tet32, tet40*) [28] (Figure 8B). The decrease in frequency observed when treatment was with amoxicillin alone was much less for most of the genes in the presence of SYN-007. Exceptions included *aph2–1b*, which increased in amoxicillin + Formulation 1 and amoxicillin + Formulation 2 resistomes, and *tet40* which was unaffected with amoxicillin + Formulation 1, and displayed a slight increase with amoxicillin + Formulation 3 (Figure 8B). In general, a larger number of resistance genes were affected and to a greater extent in the amoxicillin alone resistomes, compared to exposure to amoxicillin + SYN-007.

Results of the microbiome and resistome analyses demonstrate exposure of the canine gut microbiome to orally administered amoxicillin causes alterations in the gut microbiome composition and changes in the gut resistome, including emergence and enhancement of specific antibiotic resistance genes. Oral amoxicillin-mediated gut microbiome damage was attenuated by co-administration of SYN-007, with the three SYN-007 formulations displaying differing levels of microbiome protection.

## 4. Discussion

Inactivation of antibiotics in the GI tract represents an effective strategy to preserve microbiome composition in the colon and reduce propagation of antibiotic resistant microorganisms. Here, an oral formulation of the beta-lactamase enzyme, ribaxamase, intended to protect the gut microbiome from damage caused by orally administered beta-lactam antibiotics, was identified. Three delayed release ribaxamase formulations, SYN-007, with differing in vitro dissolution characteristics were selected for evaluation in a canine model of oral amoxicillin-mediated gut dysbiosis. An analogous porcine model was used previously to verify ribaxamase efficacy for gut microbiome protection in animals treated with the IV beta-lactam, ceftriaxone [6]. One SYN-007 formulation (Formulation 3) demonstrated attenuation of oral amoxicillin-mediated gut microbiome alteration and reduction of antibiotic resistance, without interfering with oral amoxicillin systemic absorption. This novel SYN-007 formulation, composed of EUDRAGIT^®^ L30-D55 coated enzyme pellets within EUDRAGIT^®^ FS30D coated capsules, greatly broadens ribaxamase utility to include gut microbiome protection from both IV and oral beta-lactams.

A key consideration in the design of the SYN-007 formulations was the fact that any level of premature enzyme release or leakage could not be tolerated. This is in contrast to most GI site-directed applications where the most important characteristic is that the vast majority of drug release occurs at the target site with a small amount of leakage accepted and likely inevitable [14]. Ribaxamase is extremely efficient at degrading penicillins and most cephalosporin beta-lactam antibiotics [10]. Therefore, even a minute amount of enzyme leakage in the upper small intestine is expected to degrade an orally delivered antibiotic prior to absorption, resulting in reduced systemic levels and potentially compromised infection control efficacy. Indeed, a beta-lactamase formulated for delivery to the colon by incorporation into pectin beads interfered with oral amoxicillin systemic absorption when delivered orally to mice, presumably caused by enzyme leakage in the upper small intestine [29]. This problem was ameliorated by coating the beta-lactamase-containing pectin beads with polyethylenimine to prevent premature enzyme release [30].

Here, three SYN-007 oral formulations, composed of enzyme-covered beads coated with a pH-triggered enteric polymer, were engineered for use with oral amoxicillin. As an additional safeguard to preclude enzyme leakage in the upper small intestine, Formulations 1 and 3 employed a dual coating approach composed of enteric-coated beta-lactamase pellets loaded into small, size 9h capsules (2.69 mm diameter × 5.1 mm length) covered with EUDRAGIT^®^ FS30D. Encapsulating the enteric-coated enzyme pellets within enteric-coated capsules served two purposes. First, the pellets remained together and protected until the capsule coating dissolved at pH >7.0, preventing them from becoming stuck and possibly damaged within the folds and crevasses of the upper small intestine. Second, the size 9h capsules are small enough to pass through a closed pyloric sphincter [31,32] and were selected to compensate for gastric emptying variations and to prevent prolonged retention and potential mechanical damage within the stomach. While all three formulations protected the gut microbiome from oral amoxicillin-mediated damage, only Formulation 3 did so without affecting oral amoxicillin systemic absorption. Therefore, Formulation 3 prevented premature beta-lactamase release in the upper small intestine and allowed sufficient enzyme release distal to the site of oral amoxicillin absorption to protect the gut microbiome. In contrast, Formulations 1 and 2 were associated with significantly reduced oral amoxicillin systemic absorption, indicating that the antibiotic was degraded within the GI tract prior to absorption and before reaching and harming the colonic microbiota. It remains unclear why the dual coating approach, successful with Formulation 3, failed to prevent early beta-lactamase release by Formulation 1. These two formulations differ by the enteric coating on the enzyme pellets, indicating that the L30-D55 pellets of Formulation 3 were superior to the FS30D pellets of Formulation 1. Therefore, it is possible that the FS30D-coated pellets were more fragile and/or prone to damage once released in the GI tract. The observation that Formulations 1 and 2, both containing FS30D pellets, displayed a more rapid in vitro dissolution kinetics than Formulation 3 is consistent with this hypothesis.

Oral amoxicillin caused significant changes in the canine gut microbiome that were attenuated in the presence of SYN-007. Gut microbiome alteration included significant reduction in bacterial richness, alpha diversity (the number of species and their relative abundance in each sample), and alteration of gut microbiome composition by loss of specific bacterial species and overgrowth of other taxa. Species in the phylum Firmicutes, including *Clostridioides, Blautia, Ruminococcus*, and *Lachospiraceae* were lost or greatly reduced in abundance, while increased abundance of *Bacteriodes* and *Escherichia* species was observed following exposure to amoxicillin alone. Of note was dramatic overgrowth of *E. coli* in dogs 3 and 4 in the amoxicillin alone cohort resulting in species monodomination [33] in dog 4. These results are consistent with those of a previous study demonstrating a shift in fecal microbial balance toward Gram-negative organisms, including those of the Enterobateriaceae family, in dogs treated with oral amoxicillin [34]. In contrast, SYN-007, co-administered with oral amoxicillin, protected both bacterial richness and alpha diversity, and resulted in less alteration of the microbiota composition. For example, the increase in *E coli* was not observed with SYN-007, and loss of commensal Firmicutes, including *Clostridioides, Blautia, Ruminococcus*, and *Lachospiraceae* species was attenuated. However, increased abundance of *Megamonas spp*., commensal gut bacteria present in healthy carnivores [35], was observed in most of the animals treated with SYN-007, especially in dogs from the Formulation 2 cohort. *Megamonas spp*. produce propionate [35], a short-chain fatty acid reported to possess potent anti-inflammatory properties [36], an observation meriting further study.

In addition to microbiome alteration, oral amoxicillin exposure resulted in changes to the antibiotic resistome that were attenuated in the presence of SYN-007. Several beta-lactamase genes encoding resistance, specifically to beta-lactam antibiotics, emerged following amoxicillin exposure, including predominately class A TEM beta-lactamases and several class D OXA beta-lactamases. Notably, the majority of the beta-lactamase genes were detected in the amoxicillin alone cohort, following antibiotic exposure, with more beta-lactamases encoded in the gut microbiome of dog 4 (44 enzyme variants), followed by dog 3 (13 enzyme variants), the same animals with microbiome overgrowth of *E. coli*, suggesting these genes were harbored by this species. Metagenomics sequencing data do not yet allow identification of those bacterial taxa possessing specific resistance genes. However, the data presented here are consistent with those of reports demonstrating emergence of beta-lactam resistant *E. coli* in dogs treated with oral amoxicillin [34] and amoxicillin/clavulanate [37].

In addition to beta-lactamases, other resistance gene frequencies were affected by antibiotic exposure. Interestingly, genes with increased frequencies following amoxicillin exposure, including the *emrK, acrS, baeR, cpxA*, and *acrF* genes known to be harbored by *E. coli* [28], were unaffected or displayed reduced frequencies in the SYN-007 Formulation 3 cohort. Other genes such as the *tet40, mefA, aph2–1b,* and *tet32* encoded by Gram-positive organisms [28], were greatly reduced in frequency after exposure to antibiotic alone and showed minimal change in frequency in the SYN-007 Formulation 3 cohort. These observations suggest alterations in the resistome were influenced by both direct selection of species harboring genes conferring resistance to amoxicillin and by fluctuation in their relative abundance in the gut microbiota following antibiotic exposure. Previous studies have reported that the frequency of a broad range of antibiotic resistance genes is altered after antibiotic exposure in dogs and pigs, including genes conferring resistance to antibiotics other than the administered antimicrobial agent [6,34,38,39].

## 5. Conclusions

Protection of the gut microbiome from antibiotic collateral damage is important to diminish infection with opportunistic pathogens, to mitigate emergence of antimicrobial resistance, and to maintain health. Broad-spectrum antibiotics, including the beta-lactams, are especially destructive to the gut microbiome [1,2]. Notably, oral amoxicillin is the most commonly prescribed antimicrobial [12]. In this study, an effective, delayed release formulation of ribaxamase, SYN-007, was identified that attenuated alteration of both the gut microbiome and resistome composition caused by oral amoxicillin administration, without affecting oral antibiotic systemic absorption. SYN-007 has the potential to expand microbiome protection via antibiotic inactivation for both oral and parenteral beta-lactam antibiotics and to reduce emergence of antibiotic-resistant pathogens.

## Figures and Tables

**Table 1:** SYN-007 formulations. Formulation 1 was composed of 12 Eudragit® FS30D-coated enzyme pellets within FS30D-coated size 9h capsules. Five filled and coated 9h capsules were loaded into one size 0 uncoated hard capsule for a total ribaxamase dose of 9.4 mg/size 0 capsule. Formulation 2 contained 64 FS30D-coated enzyme pellets within a size 5 uncoated capsule for a total ribaxamase dose of 10 mg/size 5 capsule. Formulation 3 was composed of 8 L30-D55-coated enzyme pellets within FS30D-coated size 9h capsules. Eight filled and coated 9h capsules were loaded into one size 0 uncoated hard capsule for a total ribaxamase dose of 10 mg/size 0 capsule.

Formulation	Enzyme Pellets	Capsule	Pellets per Capsule	Capsules per dose	Delivery Package	Schematic
1	FS30D 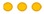	FS30D-coated Size 9h 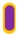	12	5	Uncoated Size 0	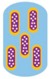
2	FS30D 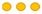	Uncoated Size 5	64	1	Uncoated Size 5	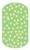
3	L30-D55 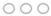	FS30D-coated Size 9h 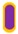	8	8	Uncoated Size 0	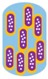

**Table 2:** Bacterial species richness of fecal microbiomes before and after amoxicillin exposure. Data are displayed as mean±SD. *p* values were obtained using the Kruskal-Walllis non-parametric ANOVA with Dunn’s Multiple Comparisons Test (GraphPad Prism 7).

Group	Bacterial Species (Mean ± SD)		Significance
	Pretreatment	Post-Treatment	*p* value
Amox Alone	307 ± 42	202 ± 3	0.006
Amox + Form 1	337 ± 61	303 ± 21	> 0.999
Amox + Form 2	274 ± 26	258 ± 29	> 0.999
Amox + Form 3	310 ± 27	259 ± 30	0.170

## Supplementary Materials

The following are available online at https://www.mdpi.com/2076-2607/7/5/150/s1, Figure S1: Principal component analysis of microbiomes of animals in all treatment groups. Figure S2: Principal component analysis of microbiomes of animals in all treatment groups except Dog 9. Figure S3: Principal component analysis of microbiomes of animals in Amoxicillin alone and Amoxicillin + Formulation 1 treatment groups with Dog 9 predose (Amoxicillin + Formulation 1). Figure S4: Heatmap analysis of the frequency of antibiotic resistance genes in the dog fecal microbiomes.

## References

[1] Crandon J.L., Nicolau D.P. (2011). Pharmacodynamic approaches to optimizing beta-lactam therapy. Crit. Care Clin..

[2] Weiss E., Zahar J.R., Lesprit P., Ruppe E., Leone M., Chastre J., Lucet J.C., Paugam-Burtz C., Brun-Buisson C., Timsit J.F. (2015). Elaboration of a consensual definition of de-escalation allowing a ranking of beta-lactams. Clin. Microbiol. Infect..

[3] Falony G., Joossens M., Vieira-Silva S., Wang J., Darzi Y., Faust K., Kurilshikov A., Bonder M.J., Valles-Colomer M., Vandeputte D. (2016). Population-level analysis of gut microbiome variation. Science.

[4] Vardakas K.Z., Trigkidis K.K., Boukouvala E., Falagas M.E. (2016). Clostridium difficile infection following systemic antibiotic administration in randomised controlled trials: A systematic review and meta-analysis. Int. J. Antimicrob. Agents.

[5] Watson T., Hickok J., Fraker S., Korwek K., Poland R.E., Septimus E. (2018). Evaluating the Risk Factors for Hospital-Onset Clostridium difficile Infections in a Large Healthcare System. Clin. Infect. Dis..

[6] Connelly S., Bristol J.A., Hubert S., Subramanian P., Hasan N.A., Colwell R.R., Kaleko M. (2017). SYN-004 (ribaxamase), an oral beta-lactamase, mitigates antibiotic-mediated dysbiosis in a porcine gut microbiome model. J. Appl Microbiol..

[7] Kokai-Kun J., Robets T., Coughlin O., Le C., Whalen H., Stevenson R., Wacher V.J., Sliman J. (2019). Use of ribaxamase (SYN-004), a beta-lactamase, to prevent *Clostridium difficile* infection in beta-lactam-treated patients: a double-blind, phase 2b, randomised placebo-controlled trial. Lancet Infect. Dis..

[8] Kokai-Kun J.F., Roberts T., Coughlin O., Sicard E., Rufiange M., Fedorak R., Carter C., Adams M.H., Longstreth J., Whalen H., Sliman J. (2017). The Oral beta-Lactamase SYN-004 (Ribaxamase) Degrades Ceftriaxone Excreted into the Intestine in Phase 2a Clinical Studies. Antimicrob Agents Chemother..

[9] Clinicaltrials.gov A study of SYN-004 for the prevention of *C. diff* in patients with a LRTI. https://clinicaltrials.gov/ct2/show/NCT02563106.

[10] Kaleko M., Bristol J.A., Hubert S., Parsley T., Widmer G., Tzipori S., Subramanian P., Hasan N., Koski P., Kokai-Kun J. (2016). Development of SYN-004, an oral beta-lactamase treatment to protect the gut microbiome from antibiotic-mediated damage and prevent Clostridium difficile infection. Anaerobe.

[11] Bristol A., Hubert S., Hofmann F., Baer H. (2017). Formulation development of SYN-004 (ribaxamase) oral solid dosage form, a beta-lactamase to prevent intravenous antibiotic-associated dysbiosis of the colon. Int J. Pharm..

[12] (2015). Prevention CfDCa: Outpatient antibiotic prescriptions—United States, 2015.

[13] Barr W.H., Zola E.M., Candler E.L., Hwang S.M., Tendolkar A.V., Shamburek R., Parker B., Hilty M.D. (1994). Differential absorption of amoxicillin from the human small and large intestine. Clin. Pharm..

[14] Philip A.K., Philip B. (2010). Colon targeted drug delivery systems: a review on primary and novel approaches. Oman. Med. J..

[15] Yoshida T., Lai T.C., Kwon G.S., Sako K. (2013). pH- and ion-sensitive polymers for drug delivery. Expert. Opin. Drug Deliv..

[16] Hubert S., Chadwick A., Wacher V., Coughlin O., Kokai-Kun J., Bristol A. (2018). Development of a Modified-Release Formulation of Lovastatin Targeted to Intestinal Methanogens Implicated in Irritable Bowel Syndrome With Constipation. J. Pharm. Sci..

[17] Nugent S.G., Kumar D., Rampton D.S., Evans D.F. (2001). Intestinal luminal pH in inflammatory bowel disease: possible determinants and implications for therapy with aminosalicylates and other drugs. Gut.

[18] Naeem M., Bae J., Oshi M.A., Kim M.S., Moon H.R., Lee B.L., Im E., Jung Y., Yoo J.W. (2018). Colon-targeted delivery of cyclosporine A using dual-functional Eudragit^®^ FS30D/PLGA nanoparticles ameliorates murine experimental colitis. Int J. Nanomed..

[19] Evonik Industries AG, Darmstadt, Germany (2012). Technical Information: EUDRAGIT L 30 D-55 and EUDRAGIT L 100–55, Summary of Safety Data. vol. TOX.LD/E. https://www.stobec.com/DATA/PRODUIT/1598~v~data_8595.pdf.

[20] Arndt M., Chokshi H., Tang K., Parrott N.J., Reppas C., Dressman J.B. (2013). Dissolution media simulating the proximal canine gastrointestinal tract in the fasted state. Eur. J. Pharm. Biopharm..

[21] Coelho L.P., Kultima J.R., Costea P.I., Fournier C., Pan Y., Czarnecki-Maulden G., Hayward M.R., Forslund S.K., Schmidt T.S.B., Descombes P. (2018). Similarity of the dog and human gut microbiomes in gene content and response to diet. Microbiome.

[22] Hasan N.A., Young B.A., Minard-Smith A.T., Saeed K., Li H., Heizer E.M., McMillan N.J., Isom R., Abdullah A.S., Bornman D.M. (2014). Microbial community profiling of human saliva using shotgun metagenomic sequencing. PLoS ONE.

[23] Ponnusamy D., Kozlova E.V., Sha J., Erova T.E., Azar S.R., Fitts E.C., Kirtley M.L., Tiner B.L., Andersson J.A., Grim C.J. (2016). Cross-talk among flesh-eating Aeromonas hydrophila strains in mixed infection leading to necrotizing fasciitis. Proc. Natl. Acad. Sci. USA.

[24] Hourigan S.K., Subramanian P., Hasan N.A., Ta A., Klein E., Chettout N., Huddleston K., Deopujari V., Levy S., Baveja R. (2018). Comparison of Infant Gut and Skin Microbiota, Resistome and Virulome Between Neonatal Intensive Care Unit (NICU) Environments. Front. Microbiol..

[25] Roy M.A., Arnaud J.M., Jasmin P.M., Hamner S., Hasan N.A., Colwell R.R., Ford T.E. (2018). A Metagenomic Approach to Evaluating Surface Water Quality in Haiti. Int. J. Environ. Res. Public Health.

[26] Shannon C.E. (1948). A mathematical theory of communication. Bell. Syst. Tech. J..

[27] Gaujoux R., Seoighe C. (2010). A flexible R package for nonnegative matrix factorization. BMC Bioinform..

[28] McArthur A.G., Waglechner N., Nizam F., Yan A., Azad M.A., Baylay A.J., Bhullar K., Canova M.J., De Pascale G., Ejim L. (2013). The comprehensive antibiotic resistance database. Antimicrob Agents Chemother..

[29] Bourgeois S., Laham A., Besnard M., Andremont A., Fattal E. (2005). In vitro and in vivo evaluation of pectin beads for the colon delivery of beta-lactamases. J. Drug Target..

[30] Bourgeois S., Tsapis N., Honnas H., Andremont A., Shakweh M., Besnard M., Fattal E. (2008). Colonic delivery of beta-lactamases does not affect amoxicillin pharmacokinetics in rats. J. Pharm. Sci..

[31] Martinez M.N., Papich M.G. (2009). Factors influencing the gastric residence of dosage forms in dogs. J. Pharm. Sci..

[32] Park H.M., Chernish S.M., Rosenek B.D., Brunelle R.L., Hargrove B., Wellman H.N. (1984). Gastric emptying of enteric-coated tablets. Dig. Dis. Sci..

[33] Taur Y., Jenq R.R., Perales M.A., Littmann E.R., Morjaria S., Ling L., No D., Gobourne A., Viale A., Dahi P.B. (2014). The effects of intestinal tract bacterial diversity on mortality following allogeneic hematopoietic stem cell transplantation. Blood.

[34] Gronvold A.M., L’Abee-Lund T.M., Sorum H., Skancke E., Yannarell A.C., Mackie R.I. (2010). Changes in fecal microbiota of healthy dogs administered amoxicillin. FEMS Microbiol. Ecol..

[35] Beloshapka A.N., Dowd S.E., Suchodolski J.S., Steiner J.M., Duclos L., Swanson K.S. (2013). Fecal microbial communities of healthy adult dogs fed raw meat-based diets with or without inulin or yeast cell wall extracts as assessed by 454 pyrosequencing. FEMS Microbiol. Ecol..

[36] Li M., van Esch B., Wagenaar G.T.M., Garssen J., Folkerts G., Henricks P.A.J. (2018). Pro- and anti-inflammatory effects of short chain fatty acids on immune and endothelial cells. Eur. J. Pharm..

[37] Schmidt V.M., Pinchbeck G., McIntyre K.M., Nuttall T., McEwan N., Dawson S., Williams N.J. (2018). Routine antibiotic therapy in dogs increases the detection of antimicrobial-resistant faecal *Escherichia coli*. J. Antimicrob Chemother..

[38] Connelly S., Subramanian P., Hasan N.A., Colwell R.R., Kaleko M. (2018). Distinct consequences of amoxicillin and ertapenem exposure in the porcine gut microbiome. Anaerobe.

[39] Looft T., Johnson T.A., Allen H.K., Bayles D.O., Alt D.P., Stedtfeld R.D., Sul W.J., Stedtfeld T.M., Chai B., Cole J.R. (2012). In-feed antibiotic effects on the swine intestinal microbiome. Proc. Natl. Acad. Sci. USA.

