# Supplementary material for: Oral Beta-Lactamase Protects the Canine Gut Microbiome from Oral Amoxicillin-Mediated Damage
Source: Microorganisms. 2019 May 27;7(5):150. doi: 10.3390/microorganisms7050150 (PMC6560916; doi:10.3390/microorganisms7050150)
Supplement: Supplementary file 1 [file microorganisms-07-00150-s001.pdf]

Supplementary Materials

Oral Beta-Lactamase Protects the Canine Gut Microbiome from Oral Amoxicillin-Mediated Damage

Sheila Connelly [1,*], Brian Fanelli [2], Nur A. Hasan [2], Rita R. Colwell [2,3] and Michael Kaleko [1]

[1] Synthetic Biologics, Inc., 9605 Medical Center Drive, Suite 270, Rockville, MD 20850, USA; mkaleko@syntheticbiologics.com
[2] CosmosID, Inc., Rockville, MD 20850, USA; brian.fanelli@cosmosid.com (B.F.), nur.hasan@cosmosid.com (N.A.H.), rita.colwell@cosmosid.com (R.R.C.)
[3] University of Maryland Institute of Advanced Computer Studies, College Park, MD 20740, USA
* Correspondence: sconnelly@syntheticbiologics.com; Tel.: +1-(240)-238–3866

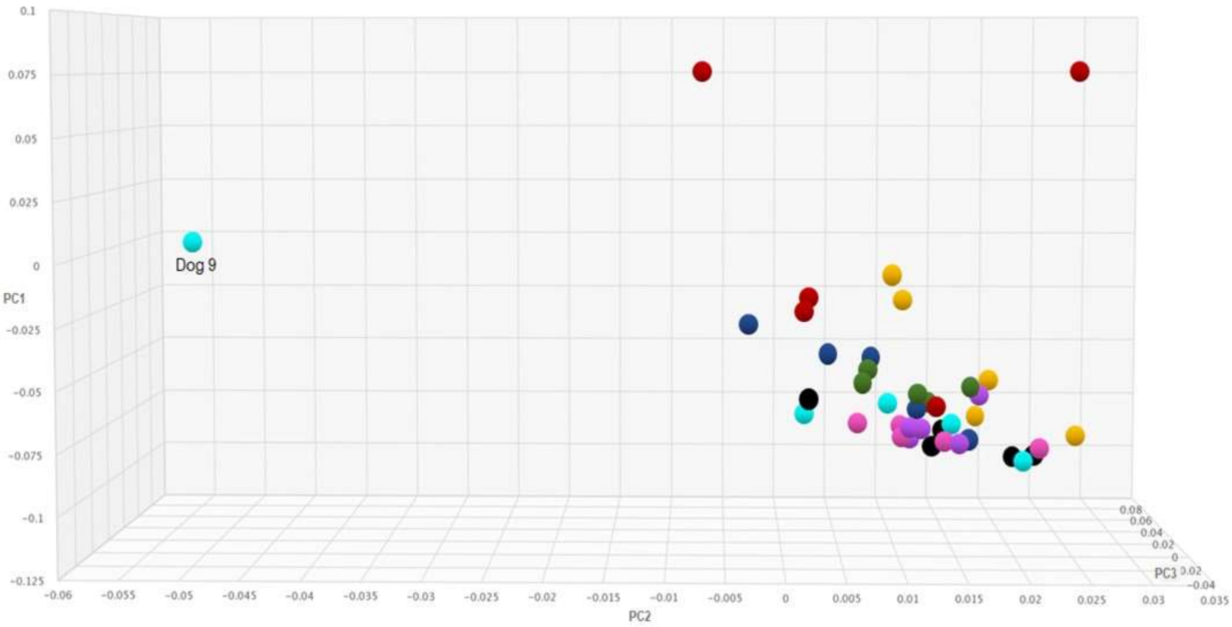

Figure S1. Principal component analysis of microbiomes of animals in all treatment groups. Black, amoxicillin pretreatment, red, amoxicillin post treatment, light blue, Amoxicillin + Formulation 1 pretreatment, dark blue, Amoxicillin + Formulation 1, post-treatment, pink, Amoxicillin + Formulation 2 pretreatment, yellow, Amoxicillin + Formulation 2 post-treatment, purple, Amoxicillin + Formulation 3 pretreatment, green, Amoxicillin + Formulation 3 post-treatment.

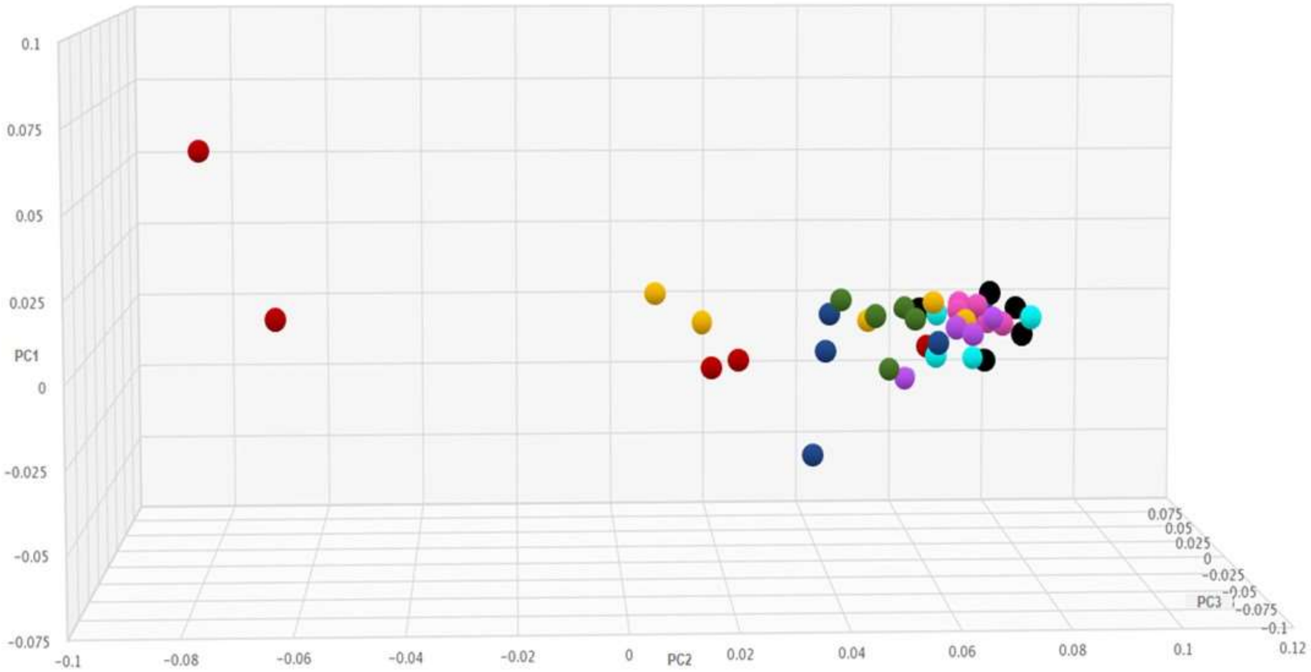

Figure S2. Principal component analysis of microbiomes of animals in all treatment groups except Dog 9. Black, amoxicillin pretreatment, red, amoxicillin post treatment, light blue, Amoxicillin + Formulation 1 pretreatment, dark blue, Amoxicillin + Formulation 1, post-treatment, pink, Amoxicillin + Formulation 2 pretreatment, yellow, Amoxicillin + Formulation 2 post-treatment, purple, Amoxicillin + Formulation 3 pretreatment, green, Amoxicillin + Formulation 3 post-treatment. Dog 9 is from the Amoxicillin + Formulation 1 cohort.

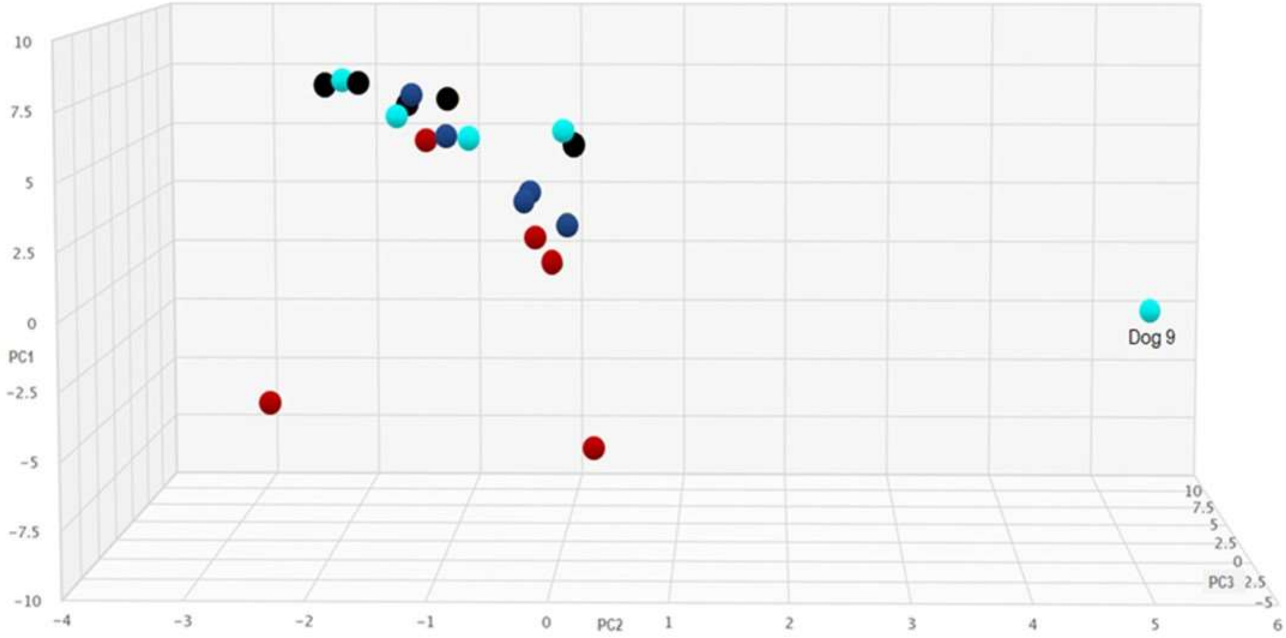

Figure S3. Principal component analysis of microbiomes of animals in Amoxicillin alone and Amoxicillin + Formulation 1 treatment groups with Dog 9 predose (Amoxicillin + Formulation 1). Compare to Figure 4B,

principal component analysis of Amoxicillin alone and Amoxicillin + Formulation 1 without Dog 9 pretreatment. Black, amoxicillin pretreatment, red, amoxicillin post treatment, light blue, Amoxicillin + Formulation 1 pretreatment, dark blue, Amoxicillin + Formulation 1, post-treatment.

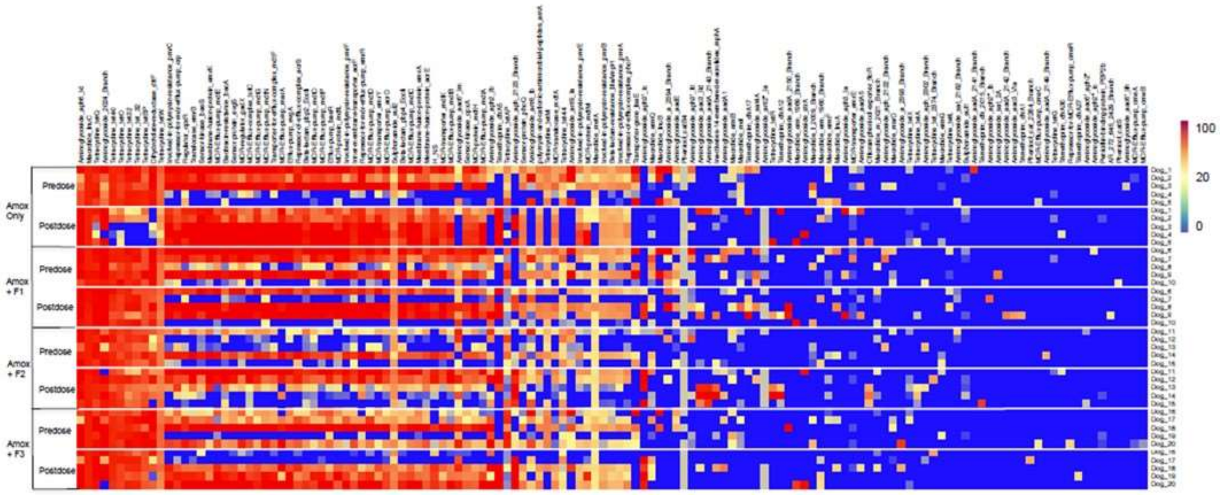

Figure S4. Heatmap analysis of the frequency of antibiotic resistance genes in the dog fecal microbiomes. Fecal microbiome metagenomics data were analyzed for the presence of antibiotic resistance genes based on the percentage gene coverage as a measure of relative gene frequency in each sample. Each row represents an individual animal at the indicated time point. Antibiotic resistance genes are displayed at the top, treatment group and day of sample collection on the left, and animal numbers on the right. The color gradient key displays a linear scale of the percent gene coverage as a measure of the relative gene frequency.
